# Evidence for Site-Specific Tunneling in H Atom Uptake at the Surface of Polyoxovanadate-Alkoxides

**DOI:** 10.1021/jacsau.6c00947

**Published:** 2026-08-19

**Authors:** M. Rebecca A. Walls, Shannon E. Cooney, Ellen M. Matson

**Affiliations:** Department of Chemistry, 6927 University of Rochester, Rochester, New York 14627, United States

**Keywords:** Polyoxometalate, Proton-coupled Electron Transfer, Tunneling, Mechanism, Metal Oxides

## Abstract

Lindqvist-type polyoxovanadate-alkoxides are used as atomically precise, soluble analogs of metal oxides to interrogate site-dependent (e.g., μ_2_-oxido vs terminal oxido) tunneling in proton-coupled electron transfer (PCET). Variable-temperature kinetic isotope effect (VT-KIE) analysis reveals that H atom uptake at the μ_2_-oxido site of [TBA]_2_[V_6_O_8_(OCH_3_)_11_] proceeds via quantum mechanical tunneling in acetonitrile, evidenced by a temperature-independent KIE, a small isotopic effect on activation energy, and positive Arrhenius prefactor ratio. This behavior is in contrast to PCET at the terminal vanadyl site of [TBA]­[V_6_O_7_(OCH_3_)_12_], which follows a semiclassical, concerted proton–electron transfer mechanism. Tunneling effects are solvent dependent; switching to dimethylformamide, a more basic solvent, results in a reduction in H-bonding interactions with the acceptor, lowering the observed rate of H atom uptake at the bridging site. This work highlights the role of site-specific basicity on tunneling in metal oxides, where competitive H-bonding with solvent disrupts tunneling.

## Graphical abstract

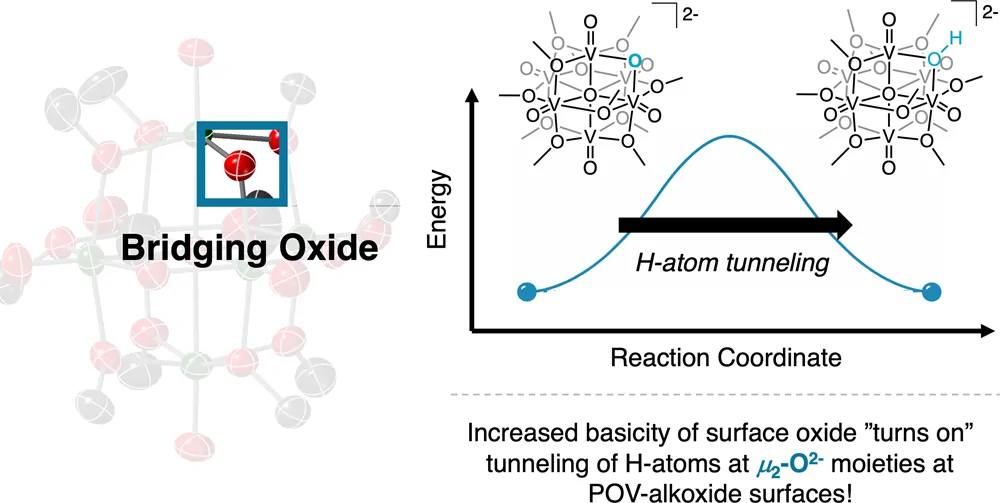

Proton-coupled electron transfer (PCET) provides a mechanistic framework for understanding how protons and electrons move in a coordinated fashion to mediate chemical transformations spanning biological redox processes, synthetic catalysis, and energy conversion.^1–6^ Within classical transition-state theory, reactions occur when thermal energy is sufficient to surmount the activation barrier separating reactants and products.^7,8^ However, the small mass of the proton allows quantum mechanical tunneling to contribute significantly to many PCET reactions, enabling transfer through rather than over the reaction barrier. Such tunneling can accelerate reaction rates, alter temperature dependences, and produce kinetic isotope effects (KIEs) that deviate from classical predictions.^9–14^ In semiclassical descriptions of PCET, electron transfer is treated quantum mechanically while proton motion is often approximated classically or through limited tunneling corrections, such that proton tunneling dominates the observed quantum effects.^15,16^ More rigorous quantum mechanical treatments describe both proton and electron motion through coupled vibronic states, permitting concerted transfer of the hydrogen atom and even tunneling by heavier isotopes such as deuterium when sufficient wave function overlap exists between reactant and product states.^12,17–19^ These effects are particularly important in systems with weak donor–acceptor coupling or dynamic structural fluctuations, including metalloenzymes, molecular electrocatalysts, and energy-conversion materials.^17,20–23^ Understanding the role of tunneling in PCET is therefore central to elucidating the mechanisms of biological and synthetic hydrogen-transfer reactions and to establishing design principles for next-generation catalysts and energy-conversion materials.

At reducible metal oxide (MOx) surfaces, PCET is frequently invoked in elementary steps involving substrate activation, oxygen atom transfer, hydrogen atom abstraction, and other charge compensation reactions, making it a central mechanistic framework for understanding interfacial reactivity.^6,16,24–26^ However, mechanistic interrogation of PCET at extended MOx remains challenging because of the structural heterogeneity of their surfaces. Indeed, the dynamic nature of MOx surfaces creates additional challenges in mechanistic assessment, where coordination environment, defects, adsorbates, and solvation can all influence proton and electron motion.^27,28^ As a result, the factors governing whether PCET proceeds through concerted or stepwise pathways and the extent to which proton transfer is classically activated or influenced by quantum mechanical effects remain poorly resolved for many MOx systems.

Developing a molecular-level understanding of PCET at metal oxide interfaces is essential for establishing structure–reactivity relationships and for the rational design of more active, selective, and mechanistically programmable MOx catalysts. To understand the mechanism of PCET at metal oxide surfaces, our group has been investigating polyoxometalates (POMs).^29–31^ Similar to MOx surfaces, POMs support communication between multiple metal centers, while their molecular nature enables in situ monitoring and atomically precise characterization of reactive intermediates — including identification of H atom uptake sites.^32–34^ To date, we have studied both Lindqvist-type polyoxovanadate-alkoxides (POV-alkoxide) and Keggin-type polyoxotungstates, using systematic structural modifications to map how surface geometry and metal-center electronics govern PCET rates and mechanisms.^34–41^ Perturbations to the surface through heteroatom substitution has been shown to allow for mechanistic control, between either stepwise or CPET mechanisms, according to changes in surface reducibility and basicity.^34,36,40,42^

A previous report from our group identified rate enhancement for H atom uptake at the surface of a POV-alkoxide localized at a bridging oxido site relative to a terminal vanadyl moiety, despite nearly identical thermodynamic driving forces and activation parameters (Figure 1; ΔG_PCET_ **1-V**_**6**_ **O**_**8**_^**2–**^ = 0.7 ± 0.1 kcal/mol; ΔG_PCET_ **2-V**_**6**_ **O**_**7**_^**1–**^ = 0.2 ± 0.1 kcal/mol).^35^ Herein, we report mechanistic investigations that indicate that the observed rate enhancement arises from quantum mechanical H atom tunneling at the bridging oxido, as confirmed by variable-temperature KIE analysis. We further investigate the impacts of H-bonding and local basicity by employing a more basic solvent, dimethylformamide (DMF), that disrupts H-bonding interactions between substrate and cluster. Eyring and variable-temperature KIE analyses of the reaction in DMF confirms the enhanced basicity at the bridging oxido promotes substrate proximity and thereby tunneling probability, which can be disrupted via solvent interactions. Together, these results demonstrate that the site of uptake in metal oxides dictates the probability of tunneling effects, where more basic bridging oxidos promote tunneling at POV-alkoxide surfaces.

**Figure 1:**
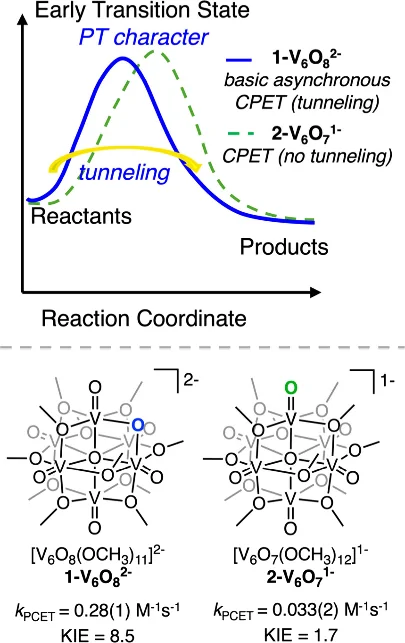
Proposed mechanistic pathways of PCET at disparate surface sites of POV-alkoxides.

## Results and Discussion

### Investigating the Origin of Rate Enhancement of PCET at Bridging Oxido

We have previously reported the observation of a 10-fold rate enhancement for H atom uptake at the bridging oxido of **1-V**_**6**_ **O**_**8**_^**2–**^ relative to that of PCET to the terminal vanadyl of **2-V**_**6**_ **O**_**7**_^**1–**^.^35^ In an attempt to elucidate the origin of these differences in reaction rates, Eyring analysis was performed for the reaction of H_2_Phen and **1-V**_**6**_ **O**_**8**_^**2–**^, revealing activation barriers (i.e., ΔG^‡^, ΔH^‡^, ΔS^‡^) similar to those reported for the reaction of H_2_Phen and **2-V**_**6**_ **O**_**7**_^**1–**^ (Scheme 1, Table 1). In both cases, the obtained values are consistent with a concerted proton electron transfer (CPET) mechanism. However, KIE values obtained for H atom uptake at **1-V**_**6**_ **O**_**8**_^**2–**^ and **2-V**_**6**_ **O**_**7**_^**1–**^ are quite different (KIE for H atom uptake at **1-V**_**6**_ **O**_**8**_^**2–**^ = 8.5; KIE for H atom uptake at **2-V**_**6**_ **O**_**7**_^**1–**^ = 1.7). The combination of comparable activation parameters but markedly different rates and KIEs suggests that classical transition-state descriptions alone may not fully account for the observed reactivity. Motivated by these discrepancies, and by analogous observations in other PCET systems where tunneling has been implicated,^13,43,44^ we sought to further investigate whether quantum mechanical effects contribute to H atom transfer at the bridging oxido site.

**Scheme 1:**
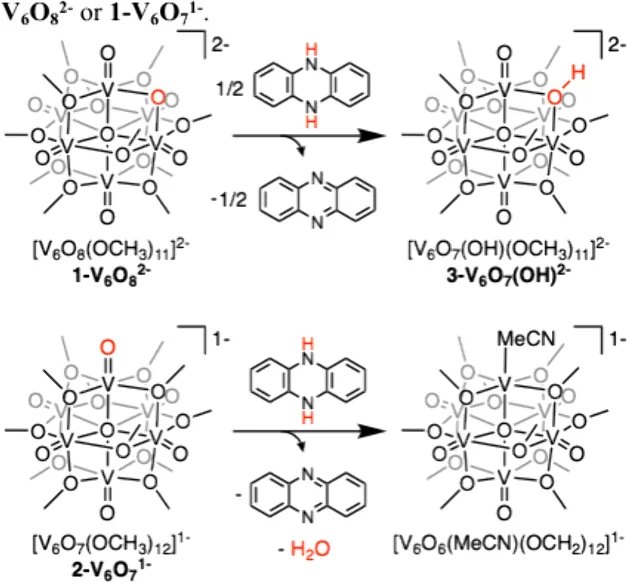
H-Atom Transfer Reactions from H_2_Phen to **1-V**_**6**_ **O**_**8**_^**2–**^ or **1-V**_**6**_ **O**_**7**_^**1–**^

**Table 1:** Kinetic and Thermodynamic Parameters of PCET from H_2_Phen to **1-V**_**6**_ **O**_**8**_^**2–**^ and **2-V**_**6**_ **O**_**7**_^**1–**^ at 248 K in MeCN^34^

	**1-V_6_O_8_^2–^**	**2-V_6_O_7_^1–^**
Rate (M^–1^ s^–1^)	0.23 ± 0.01	0.033 ± 0.002
ΔG_PCET_ (kcal/mol)	0.7 ± 0.1	0.2 ± 0.1
KIE	8.5 ± 0.1	1.7 ± 0.1
ΔG^⧧^ (kcal/mol)	16.4 ± 0.8	16.6 ± 1.8
ΔH^⧧^ (kcal/mol)	6.3 ± 0.4	6.6 ± 0.8
ΔS^⧧^ (cal/(mol K))	–34.0 ± 1.5	–33.6 ± 3.3

Transfer of proton/electron equivalents in CPET reactions can also be viewed from a quantum mechanical perspective, wherein the proton and electron are transferred through coupled vibronic states and H atom tunneling may contribute significantly to the overall reaction rate.^13,45–47^ Indeed, Hammes-Schiffer and co-workers have demonstrated that a full quantum mechanical treatment of both proton and electron motion is required to accurately describe the kinetics of many PCET reactions^12,17–19^ In such cases, H atom tunneling is invoked, reshaping the effective reaction coordinate, altering kinetic behavior and complicating conventional interpretations of structure–reactivity relationships. Under a quantum mechanical treatment, the D atom can similarly undergo tunneling effects that results in less significant KIEs (KIE < 10). As we observe both rate enhancement and a large KIE in the reaction of H_2_Phen (donor) with **1-V**_**6**_ **O**_**8**_^**2–**^ (acceptor), we hypothesized that tunneling may be involved in H atom transfer to the bridging oxide moiety at the surface of **1-V**_**6**_ **O**_**8**_^**2–**^. In contrast, we posed that CPET from H_2_Phen to **2-V**_**6**_ **O**_**7**_^**1–**^ would be more aptly treated semiclassically, with a KIE resulting from the difference from zero point energies (ZPEs) of the H/D atoms, without a significant occurrence of proton tunneling.

To verify the extent of tunneling to the disparate POV-alkoxide surface sites (e.g., bridging vs terminal oxide moieties), variable temperature KIE experiments were performed. This form of analysis has been described as the best method to distinguish between semiclassical and quantum mechanical tunneling effects.^13,48,49^ VT-KIE experiments provide insights into the isotope impact on the Arrhenius parameters of activation energy (*E*_a_) and Arrhenius prefactor (A).^13,14,49,50^ A plot of the ln­(KIE) against 1/*RT* (where R is the gas constant, and T is the temperature in Kelvin) results in a line with a slope reflecting the isotopic effect on *E*_a_ (slope = Δ*E*_a_ = *E*_a,D_ – *E*_a,H_), and a y-intercept reflecting the isotopic impact on the reaction prefactor, A (y-intercept = ln­(A_H_/A_D_). In the case of quantum mechanical tunneling, the KIE is expected to be largely temperature independent, resulting in a small isotopic effect on Δ*E*_a_ and a more significant impact on A. In other words, a small Δ*E*_a_ is caused by small observed differences between the activation energies due to tunneling of both H- and D atoms, and only changes to the collision rate are significantly impacted by the temperature.

KIEs of PCET from 5,10-dihydro- or 5,10-dideutero-phenazine (H_2_Phen and D_2_Phen, respectively) to **1-V**_**6**_ **O**_**8**_^**2–**^ and **2-V**_**6**_ **O**_**7**_^**1–**^ were investigated in acetonitrile (MeCN) across a temperature range of −35 to 25 °C.^35^ Briefly, reductant was injected into a solution of POV-alkoxide normalized to a given temperature, and reaction progress was monitored by electronic absorption spectroscopy (UV–vis).^35–38^ The observed rates (*k*_*obs*_) of H/D atom uptake were normalized by reductant concentration and a probability factor, which accounts for the number of H­(D)-atoms available in the substrate (2 H·/D·) and the number of possible sites for uptake (i.e., 6 V = O sites in **2-V**_**6**_ **O**_**7**_^**1–**^ and 1 V–O–V site in **1-V**_**6**_ **O**_**8**_^**2–**^) to yield to rate constant *k*_PCET_. This method accounts for transfer of either H atom (1st or second) as well as available sites of H atom uptake, as previously reported by our group.^35,37,42,51–53^ Full details of the experiment and data analysis can be found in the Supporting Information.

A plot of ln­(KIE) as a function of 1/*RT* provides insight into the isotopic impacts on Δ*E*_a_ and A (Figure 2, Figures S1–S4). In the case of H atom uptake at a terminal vanadyl moiety of **2-V**_**6**_ **O**_**7**_^**1–**^, the positive slope of 1.90 kcal/mol is approximately equal to the energy difference associated with the change in ZPEs of typical O–H/O–D systems (∼2 kcal/mol).^54^ This observation is consistent with a semiclassical treatment, wherein the KIE emerges as a result of differences in ZPEs of the O–H and O–D bonds. Additionally, the negative y-intercept (ln­(A_H_/A_D_) = −3.30) is consistent with systems where semiclassical tunneling, or no tunneling, is observed.^13^ Coupled with the reported KIE of 1.7, we conclude that insignificant tunneling, if any, is operative in PCET from H_2_Phen for PCET to **2-V**_**6**_ **O**_**7**_^**1–**^. Thus, the mechanism of H atom transfer to **2-V**_**6**_ **O**_**7**_^**1–**^ is dictated by TS barrier heights and respective ZPEs.

**Figure 2:**
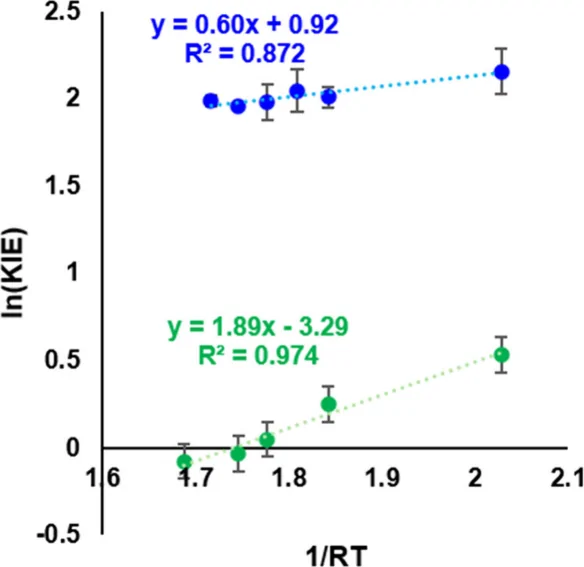
Plots of ln­(KIE) vs 1/*RT* for the reactions of **1-V**_**6**_ **O**_**8**_^**2–**^ (blue) and **2-V**_**6**_ **O**_**7**_^**1–**^ (green) with H_2_Phen and D_2_Phen in MeCN.

In the case of H-atom transfer from H_2_Phen to **1-V**_**6**_ **O**_**8**_^**2–**^, a significantly smaller temperature dependence on KIEs is observed (Δ*E*_a_ = 0.60 kcal/mol). This limited change in KIE over a range of temperatures is consistent with results described previously for H-atom transfer reactions dictated by quantum mechanical tunneling effects (Table 2).^13,43,44,55–58^ The y-intercept value, ln­(A_H_/A_D_) = 0.92, is positive, reflecting changes in collision frequencies in this reaction as a function of temperature, where H-atoms collide more frequently than D-atoms. This would be consistent with increased proximity of the H-atom donor and acceptor and the bridging oxide of **1-V**_**6**_ **O**_**8**_^**2–**^, allowing for more facile tunneling of the H-atom over the D-atom. These values together suggest that the rate enhancement of H-atom transfer from H_2_Phen to **1-V**_**6**_ **O**_**8**_^**2–**^ is due to proton/electron tunneling together as a single particle.

**Table 2:** Isotope Impact on Arrhenius Parameters for Quantum Mechanical Tunneling in H-Atom Transfer Reactions

Reaction	Δ*E*_a_	ln(A_H_/A_D_)	ref
**1-V_6_O_8_^2–^** + H_2_Phen	0.6(1)	0.9(2)	*This work*
PhB(^t^BuIm)_3_Co^III^O + Fluorene	0.28(8)	0.8(2)	^13^
LCu^III^OH + DHA^a^	0.4(2)	3(3)	^44^
4-oxo-TEMPO^•^ + TEMPOH^b^	0.3(6)	1.1(6)	^55^
SLO^c^	0.9(2)	2.9(3)	^57^
PHM^d^	0.4(3)	1.8(5)	^58^

a Dihydroanthracene.

b Values reported in DCM, with similar results reported in MeCN.

c Soybean lipoxygenase-I.

d Peptidylglycine a-hydroxylating monooxygenase.

To explain this observation, we consider the inherent differences of the sites of H-atom uptake in **1-V**_**6**_ **O**_**8**_^**2–**^ and **2-V**_**6**_ **O**_**7**_^**1–**^, that is an oxido bridged by 2 V centers and a terminal vanadyl oxygen, respectively. Bridging oxido groups at the surface of polyoxometalates are traditionally considered more nucleophilic than the terminal M=O moieties. Indeed, our group has observed this phenomenon across the range of POMs we have investigated in PCET reactions.^35,59–61^ Additionally, the bridging oxido would be expected to have fewer degrees of freedom due to anchoring at two V sites, which can enhance tunneling by lowering reorganization energies.^47,62^ As such, H-atom transfer between H_2_Phen and **1-V**_**6**_ **O**_**8**_^**2–**^ likely proceeds through the formation of a strongly associated donor–acceptor pair. Access to this H-bonded “transition state” results in a lower reorganization energy due to localization of the proton donor to the acceptor site, which increases the quantum uncertainty of the momentum in the wave function, enhancing the probability of H-atom tunneling.^45,47^ In contrast, the terminal vanadyl of **2-V**_**6**_ **O**_**7**_^**1–**^ is less basic, likely translating to the formation of weaker contact pairs with H_2_Phen, with larger reorganization energies hindering tunneling effects.

As described above, we propose that the tunneling observed at **1-V**_**6**_ **O**_**8**_^**2–**^ is attributed to a more basic surface promoting a tighter donor–acceptor pair. This hypothesis suggests that H-atom uptake at **1-V**_**6**_ **O**_**8**_^**2–**^ might proceed through a basic asynchronous CPET pathway. Traditionally, asynchronicity in PCET is attributed to individual contributions from the relevant intermediates in stepwise pathways, where the proton and electron are treated as separate entities based on a semiclassical theory.^7,8,45,63^ In this semiclassical treatment, tunneling of the proton enhances the rate and generates a large KIE (>10) as a result of mass-transfer dynamics and changes in zero-point energies (ZPEs) for the proton and deuteron. Discrepancies in the rate of H- and D-atom transfer across temperatures is often observed, translating to temperature-dependent KIE values for the reaction.^13,45,64^ For example, in basic asynchronous C–H activations, many metal complexes exhibit more semiclassical tunneling, with KIEs greater than 10, with positive slopes, greater than the standard ZPE difference of 1.4 kcal/mol, and a negative y-intercept.^14^ However, some basic asynchronous PCET at cobalt-oxo and copper-oxo systems have displayed similarly small slopes with positive y-intercepts, attributed to quantum mechanical tunneling (Table 2).^13,43,44^ This is more similar to the tunneling observed in enzymatic systems, which may be due to a close contact between the reactants providing a shorter tunneling distance, as would be observed in a tight active site of an enzyme. As such, the localization of reactivity, and enhanced basicity at **1-V**_**6**_ **O**_**8**_^**2–**^ lend itself toward a tunneling mechanism that is driven by the differences in basicity of substrate and cluster driving interactions between each.

Notably, the mechanistic shift observed in PCET at the bridging oxido of **1-V**_**6**_ **O**_**8**_^**2–**^ suggests that H-atom uptake is site-dependent in these systems. Despite identical driving forces, tunneling is limited to the bridging oxido site due to the enhanced basicity of the reactive site generates a tighter contact-pair that allows for tunneling; however, the terminal vanadyl cannot access this reactivity, leading to a kinetic regioselectivity for the bridging site.^35^ This may provide a new handle for designing materials, as both mechanistic shifts and kinetic advantages may be leveraged for product selectivity or catalytic performance.

### Solvent Dependent Tunneling Effects

Interactions with the solvent (e.g., dipole–dipole, H-bonding, etc.) are an important consideration in determining the mechanisms of PCET reactions and facilitation of tunneling, impacting the total reorganization and potential energy surfaces.^9,65–69^ For example, solvent based H-bonding interactions can impact the proximity of interactions at the uptake site, which promotes quantum mechanical H-atom tunneling.^55,65,70,71^ This is particularly relevant to this work, as we hypothesize that the tunneling observed at **1-V**_**6**_ **O**_**8**_^**2–**^ arises from increased H-bonding interactions between substrate and the nucleophilic bridging oxide site. As such, to further probe the site-dependent tunneling effects at **1-V**_**6**_ **O**_**8**_^**2–**^ we repeated the VT-KIE analysis in DMF. DMF is a more basic solvent in comparison to MeCN, which we hypothesized would disrupt the H-bonding between H_2_Phen and **1-V**_**6**_ **O**_**8**_^**2–**^, thereby interrupting the tunneling pathway.

Initially, we attempted to investigate the rate of H-atom uptake via identical pseudo-first order kinetic analysis, at −25 °C. However, the observed rate was significantly slower than that observed in MeCN, causing H_2_O condensation to obscure the accurate reaction progression, prompting us to shift to a higher temperature for reaction analysis (25 °C). Plotting the observed rates against respective H_2_Phen concentrations (6–10 mM) provides the rate of the reaction in the slope. To obtain the rate of PCET (k_PCET_), the slope is normalized for the probability of H-atom transfer (2) for the 2 H-atoms of H_2_Phen to the single briding oxido site, revealing a k_PCET_ of 0.5 ± 0.1 M^–1^s^–1^ (Figure 3; Figure S5). This is an order of magnitude lower than in MeCN (k_PCET_ = 2.49 M^–1^ s^–1^). Notably, the rate collected for D_2_Phen and **1-V**_**6**_ **O**_**8**_^**2–**^ at this temperature reveals a KIE of 2.5, as opposed to 7.2 in MeCN. This value is more consistent with that reported for **2-V**_**6**_ **O**_**7**_^**1–**^ in MeCN, where tunneling is not observed. To investigate the overall solvent effect, PCET from excess H_2_Phen to **2-V**_**6**_ **O**_**7**_^**1–**^ in DMF was investigated under the same conditions. In the case of **2-V**_**6**_ **O**_**7**_^**1–**^, the probability of H-atom transfer (12) accounts for the 6 possible terminal vanadyl sites for H-atom uptake by the 2 H-atoms of H_2_Phen, revealing a k_PCET_ of 0.55 ± 0.06 M^–1^s^–1^ (Figure 3; Figure S6). We note that this rate is similar to that observed in MeCN, suggesting that solvent viscosity does not impart a large kinetic effect on the rate of H-atom uptake

**Figure 3:**
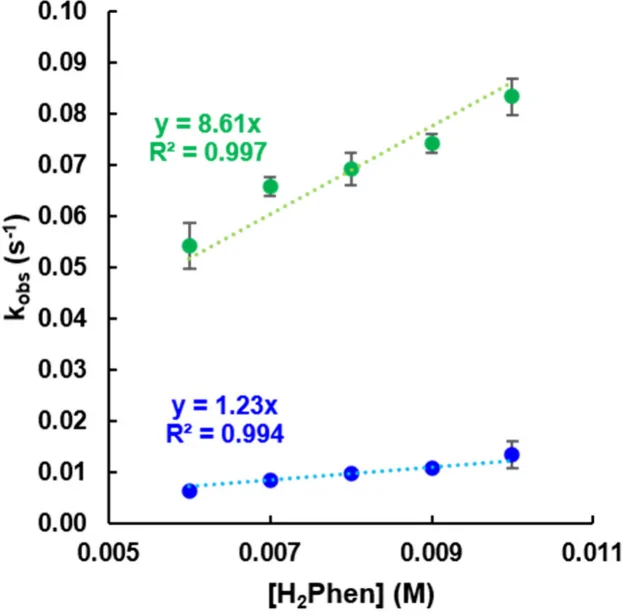
Pseudo-first order kinetic traces of PCET from H_2_Phen (6–10 mM) to **1-V**_**6**_ **O**_**8**_^**2–**^ (blue, 0.6 mM), and **2-V**_**6**_ **O**_**7**_^**1–**^ (green, 0.6 mM) in DMF at 25 °C.

The above results suggest that the suppression of the rate of H-atom uptake observed at **1-V**_**6**_ **O**_**8**_^**2–**^ in DMF is not a product of solvent viscosity, but instead solvent basicity, suggesting that the disruption to H-bonding between substrate and cluster prevents H-atom tunneling. Indeed, reports of solvent polarity and H-bonding abilities impact on tunneling include radical H-atom transfer reactions and C–H activation reactions. For example, in the pseudo self-exchange of H-atoms between hydroxylamine and nitroxyl radicals, or phenol and free radical, tunneling effects are disrupted by H-bonding solvents.^55,72–74^ In this work, it is noted that DMF and MeCN have similar relative polarities, dielectric constants, and dipole moments.^75^ The main solvent parameter impacting tunneling reactivity in this system can be summarized by the H-bonding basicity factor (β), which is twice as large in DMF (β = 0.69) relative to MeCN (β = 0.37).^76^ We hypothesize that this particular solvent parameter influences H-bonding interactions essential for tunneling from H_2_Phen to **1-V**_**6**_ **O**_**8**_^**2–**^.

For mechanistic insights, kinetics of each cluster with excess H_2_Phen were monitored across a range of temperatures (5–25 °C; Figure 4; Figures S7 and S8). Small changes are observed in the enthalpic and entropic contributions to the transition state of PCET from H_2_Phen to the POV-alkoxides in DMF, relative to the same reactions in MeCN. This is consistent with changes to the solvent reorganization in the transition state predicted when switching to DMF as a function of the change in viscosity. The small changes in ΔH^‡^ and ΔS^‡^ compensate one another, resulting in equivalent ΔG^‡^ values for PCET to both **1-V**_**6**_ **O**_**8**_^**2–**^ and **2-V**_**6**_ **O**_**7**_^**1–**^ in DMF (Figure 4, Table 3).^35,53^ However, Eyring analysis may not fully reflect the reaction progression profile if tunneling is dominant, as observed in MeCN, where identical barriers are observed between **1-V**_**6**_ **O**_**8**_^**2–**^ and **2-V**_**6**_ **O**_**7**_^**1–**^, but significant tunneling is only observed at **1-V**_**6**_ **O**_**8**_^**2–**^.

**Table 3:** Kinetic and Thermodynamic Parameters of PCET from H_2_Phen to **1-V**_**6**_ **O**_**8**_^**2–**^ and **2-V**_**6**_ **O**_**7**_^**1–**^ at 298 K in DMF

	**1-V_6_O_8_^2–^**	**2-V_6_O_7_^1–^**
Rate (M^–1^ s^–1^)	0.5 ± 0.1	0.55 ± 0.06
KIE	2.5 ± 0.2	1.3 ± 0.1
ΔG^⧧^ (kcal/mol)	17.8 ± 0.7	17.5 ± 1.7
ΔH^⧧^ (kcal/mol)	8.5 ± 0.4	7.4 ± 0.8
ΔS^⧧^ (cal/(mol K))	–31.1 ± 1.2	–34.0 ± 2.9

**Figure 4:**
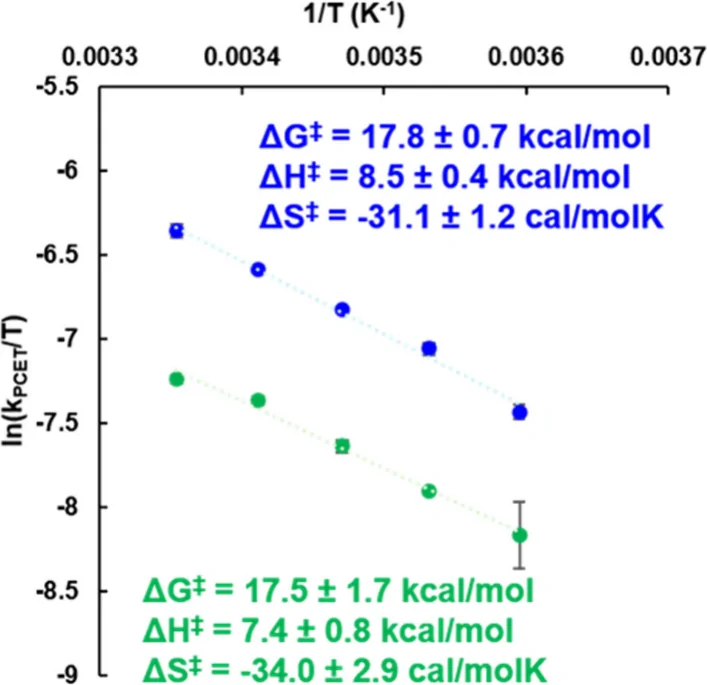
Eyring analysis of PCET from H_2_Phen (6 mM) to **1-V**_**6**_ **O**_**8**_^**2–**^ (0.6 mM, blue) and **2-V**_**6**_ **O**_**7**_^**1–**^ (0.6 mM, green) in DMF at 5–25 °C. Activation parameters calculated are provided, with G calculated at 298 K.

To parse the solvent impact on tunneling effects, particularly at **1-V**_**6**_ **O**_**8**_^**2–**^, VT-KIE analysis was performed in DMF. The plot of ln­(KIE) vs 1/*RT* reveals a positive slope for **1-V**_**6**_ **O**_**8**_^**2–**^, and a large, negative y-intercept (ln­(A_H_/A_D_) = −17(3); Figure 5, Figures S9 and S10). The positive slope (Δ*E*_a_ = 11(1) kcal/mol) reflects significant changes between activation of H- and D- transfer. With a Δ*E*_a_ greater than the standard difference in ZPEs (<2 kcal/mol), this implies some enhanced transfer of the H^+^, or semiclassical tunneling effects, where tunneling of the H^+^ is allowed, while D^+^ tunneling is not. Indeed, as we approach lower temperatures, the observed KIE approaches a KIE magnitude typically attributed to proton-only tunneling effects, with a KIE of 9.5 at 5 °C.^10,12,13^

**Figure 5:**
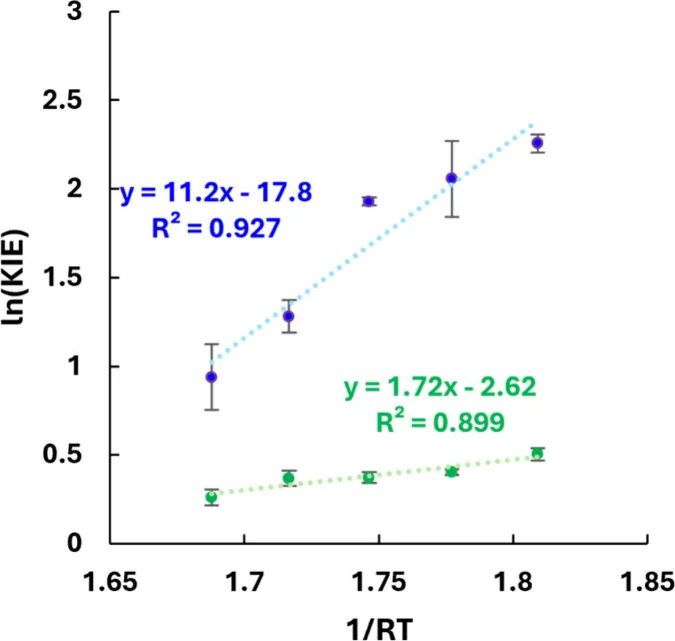
Plots of ln­(KIE) vs 1/*RT* for the reactions of **1-V**_**6**_ **O**_**8**_^**2–**^ (blue) and **2-V**_**6**_ **O**_**7**_^**1–**^ (green) with H_2_Phen and D_2_Phen in DMF.

In this case, due to the dominating effects of H^+^ tunneling, the observed changes in *E*_a_ and ln­(A_H_/A_D_) are exaggerated due to the change in temperature dependence between the tunneled H^+^ and classical progression of the deuteron. These effects have been seen in other systems, where significantly positive slopes and negative y-intercepts coincide with semiclassical proton tunneling.^77–80^ Notably, the slope and intercept are of a larger magnitude than most reported cases. The significant suppression of rate of H atom uptake at **1-V**_**6**_ **O**_**8**_^**2–**^, and transition to a semiclassical tunneling regime, indicates that the tunneling effects are significantly impacted by the basicity of the solvent. In terms of basicity and H-bonding strength, summarized in the term β, DMF more readily forms stronger H-bonds than MeCN. This propensity toward H-bonding lends to DMF disrupting the arrangement of substrate and cluster that allows for facile tunneling.

On the other hand, when VT-KIE experiments are performed to assess the mechanism of H atom transfer from H_2_Phen to **2-V**_**6**_ **O**_**7**_^**1–**^ in DMF, minimal changes to the Δ*E*_a_ are observed (MeCN = 1.9(1), DMF = 1.7(3); Figure 5, Figures S11 and S12). This observation suggests that in the case of H atom uptake at the terminal oxide of **2-V**_**6**_ **O**_**7**_^**1–**^, solvent does not have a major impact on the mechanism of CPET. Indeed, the mechanism of H atom uptake at **2-V**_**6**_ **O**_**7**_^**1–**^ is thought to proceed through a semiclassical CPET mechanism, as has been proposed for **2-V**_**6**_ **O**_**7**_^**1–**^, with the KIE originating from the ZPE difference. Similarly, the negative y-intercept, or ln­(A_H_/A_D_), is of a similar magnitude (MeCN = −3.3(3), DMF = −2.6(5)) consistent with minimal semiclassical proton to no tunneling effects playing a role in the reaction progression.

Whereas minimal changes to the reaction profile of **2-V**_**6**_ **O**_**7**_^**1–**^ as a function of solvent, the rate of H atom uptake at **1-V**_**6**_ **O**_**8**_^**2–**^ and tunneling effects are significantly impacted by the change in solvent. According to the lack of change in Eyring parameters, there does not appear to be any shift in the mechanism of PCET as a function of solvent. However, in DMF the solvent can compete with the cluster surface for H-bonding with the substrate, disrupting the probability of tunneling at **1-V**_**6**_ **O**_**8**_^**2–**^. As such, more of the reaction likely proceeds through the TS, resulting in consistent rates between **1-V**_**6**_ **O**_**8**_^**2–**^ and **2-V**_**6**_ **O**_**7**_^**1–**^ in DMF.

## Conclusions

According to a previous report from our group noting concurrent rate enhancement and increased KIE at the bridging oxido relative to the terminal oxido moiety, we sought to elaborate on the factors dictating site-dependent PCET reactivity in POV-alkoxides. In particular, the extent of tunneling in the H atom transfer from H_2_Phen to the bridging oxido of **1-V**_**6**_ **O**_**8**_^**2–**^ and terminal oxido of **2-V**_**6**_ **O**_**7**_^**1–**^ were compared via VT-KIE analysis. This method has been reported to provide insights into tunneling effects via relative changes in the activation energy and Arrhenius prefactors. This report reveals that extensive quantum mechanical tunneling is observed at the bridging oxido of **1-V**_**6**_ **O**_**8**_^**2–**^, reflected in the small Δ*E*_a_ (0.6(1) kcal/mol) and positive y-intercept (ln­(A_H_/A_D_) = 0.9(2)), reflecting the temperature dependence of collision rates leading to the small shifts in KIE as a function of temperature. On the other hand, PCET to the terminal oxido exhibits a more traditional KIE (1.7), as well as a Δ*E*_a_ (1.9 kcal/mol) consistent with traditional ZPE differences between H atom and D atoms (>2 kcal/mol). Collectively, the KIE and Δ*E*_a_ suggest minimal to no tunneling effects at the terminal oxido. The increased local basicity of the bridging oxido promotes proximity between substrate and cluster, allowing for more facile tunneling.

To probe the local surface impacts, we sought to interrupt the probability of tunneling by disrupting any H-bonding interactions between substrate and cluster through H-bonding interactions with a more basic solvent, DMF. In this case, the rate of H atom uptake at both **1-V**_**6**_ **O**_**8**_^**2–**^ and **2-V**_**6**_ **O**_**7**_^**1–**^ are equivalent, and consistent with that reported for **2-V**_**6**_ **O**_**7**_^**1–**^ in MeCN. This suggests the solvent viscosity does not play a major role in dictating the kinetic impacts at **1-V**_**6**_ **O**_**8**_^**2–**^. Additionally, the relative polarity and dielectric constants are similar for both MeCN and DMF, such that the major solvent parameter contributing to changes in reactivity is the basicity and H-bonding capacity, β. VT-KIE analysis confirms minimal changes at **2-V**_**6**_ **O**_**7**_^**1–**^ relative to the MeCN profile, confirming minimal tunneling and no mechanistic shift at **2-V**_**6**_ **O**_**7**_^**1–**^ as a function of solvent. On the other hand, where extensive tunneling at **1-V**_**6**_ **O**_**8**_^**2–**^ is noted in MeCN, in DMF the VT-KIE reveals a high temperature dependence, greater than expected for a system dependent on ZPE differences. Instead, the large positive Δ*E*_a_ and large negative ln­(A_H_/A_D_) suggests semiclassical proton tunneling, where the deuteron does not as readily tunnel as the proton. Overall, this work highlights site-dependent tunneling effects in metal-oxides that can be modulated with local solvent interactions.

## Supplementary Material



## Notes

Kinetic traces of all reactions and Arrhenius plots (PDF)
