# Supplementary material for: Evidence for Site-Specific Tunneling in H Atom Uptake at the Surface of Polyoxovanadate-Alkoxides
Source: JACS Au. 2026 Aug 19;6(9):5287–94. doi: 10.1021/jacsau.6c00947 (PMC13625538; doi:10.1021/jacsau.6c00947)
Supplement: Supplementary file 1 [file au6c00947_si_001.pdf]

Supporting Information

Evidence for Site-specific Tunneling in H-atom Uptake at the Surface of Polyoxovanadate-alkoxides

M. Rebecca A. Walls, Shannon E. Cooney, and Ellen M. Matson*

Department of Chemistry, University of Rochester, Rochester, New York 14627, USA

Supporting Information Table of Contents.

Experimental Section S2

Figure S1. Variable temperature (248-293 K) kinetic traces of **1-$V_6O_8^{2-}$** and excess H_2Phen in MeCN....... S5

Figure S2. Variable temperature (248-293 K) kinetic traces of **1-$V_6O_8^{2-}$** and excess D_2Phen in MeCN....... S6

Figure S3. Variable temperature (273-293 K) kinetic traces of **2-$V_6O_7^{1-}$** and excess D_2Phen in MeCN....... S7

Figure S4. Arrhenius plot of PCET from H_2Phen and D_2Phen to **1-$V_6O_8^{2-}$** in MeCN from -25 to 25 °C........ S8

Figure S5. Psuedo-first order kinetic traces of **1-$V_6O_8^{2-}$** and excess H_2Phen in DMF at 25 °C. S9

Figure S6. Psuedo-first order kinetic traces of **2-$V_6O_7^{1-}$** and excess H_2Phen in DMF at 25 °C. S10

Figure S7. Variable temperature (278-298 K) kinetic traces of **1-$V_6O_8^{2-}$** and excess H_2Phen in DMF. S11

Figure S8. Variable temperature (278-298 K) kinetic traces of **2-$V_6O_7^{1-}$** and excess H_2Phen in DMF. S12

Figure S9. Variable temperature (278-298 K) kinetic traces of **1-$V_6O_8^{2-}$** and excess D_2Phen in DMF. S13

Figure S10. Arrhenius plot of PCET from H_2Phen and D_2Phen to **1-$V_6O_8^{2-}$** in DMF from 5 to 25 °C. S14

Figure S11. Variable temperature (278-298 K) kinetic traces of **2-$V_6O_7^{1-}$** and excess D_2Phen in DMF. S15

Figure S12. Arrhenius plot of PCET from H_2Phen and D_2Phen to **2-$V_6O_7^{1-}$** in DMF from 5 to 25 °C. S16

References S17

Experimental Section

General Considerations. All chemical manipulations were carried out in the absence of water and oxygen using standard Schlenk techniques or in a UniLab MBraun inert atmosphere drybox under a dinitrogen atmosphere. All glassware was oven-dried for a minimum of 4 h and cooled in an evacuated antechamber prior to use in the drybox. Solvents were dried and deoxygenated on a glass contour system (Pure Process Technology, LLC) and stored over 3 Å molecular sieves purchased from Fisher Scientific and activated prior to use. All solvents were assessed prior to experiments using a Karl Fisher Titration apparatus, confirming <5 ppm water content. 9,10-phenazine was purchased from Millipore Sigma and used as received. POV-alkoxides $[^nBu_4N]_2[V_6O_8(OCH_3)_{11}]$ (**1-$V_6O_8^{2-}$**)[1] and $[^nBu_4N][V_6O_7(OCH_3)_{12}]$ (**2-$V_6O_7^{1-}$**)[2] were prepared according to previously reported procedures. 9,10-dihydrophenazine (H_2Phen)[3] and 9,10-dideuterophenazine-d_2 (D_2Phen)[4] were generated following literature precedent.

^{1}H NMR spectra were recorded at 500 MHz on a Bruker DPX-500 spectrometer, locked on the signal of deuterated solvents.[5] All chemical shifts were reported relative to the peak of the residual H signal in deuterated solvents. CD_3CN was purchased from Cambridge Isotope Laboratories, degassed by three freeze–pump–thaw cycles, and stored over fully activated 3 Å molecular sieves.

Electronic absorption measurements were recorded at room temperature in anhydrous acetonitrile (MeCN) or dimethylformamide (DMF) in a sealed 1 cm quartz cuvette with an Agilent Cary 60 UV-vis spectrophotometer or an Agilent Cary 3500 spectrophotometer. Kinetic experiments were carried out on an Agilient Cary 60 UV-vis spectrophotometer held at desired temperatures using an Unisoku CoolSpek UV cryostat, as well as an Agilent Cary 3500 UV-vis spectrophotometer held at desired temperatures with an integrated Peltier temperature control system.

General Procedures for Variable Temperature Kinetic Analyses

Pseudo-first-order reaction conditions were used to establish the variable temperature kinetic isotope effects between the POV clusters **1-$V_6O_8^{2-}$** and **2-$V_6O_7^{1-}$** (0.6 mM) with H/D-atom transfer reagents H_2Phen and D_2Phen (6 mM). Rates for variable temperature H-atom transfer from H_2Phen to **2-$V_6O_7^{1-}$** were previously reported.[6] Using a UV-vis spectrophotometer with temperature controls set to desired temperature (248-303 K), reactions between cluster and substrate were tracked by monitoring the absorbance at 1025 nm over the reaction coordinate. Samples of cluster stock solutions in MeCN or DMF were loaded in a long-necked quartz cuvette and sealed with a rubber septum and sealed with electrical tape before removing from the glovebox. In a 1 mL syringe (21G needle), a sample of reductant stock solution was measured prior to removal from the glovebox. After equilibrating to the set temperature in the spectrophotometer, data acquisition began, and the reductant solution was forcefully injected to ensure efficient sample mixing, as well as stirring over the course of the reaction. Upon the completion

of the reaction, the plot of absorbance over time was fit to the following equation by least squares fitting:

$$A_t = A_{\text{inf}} + (A_0 - A_{\text{inf}})e^{-k_{\text{obs}} t} \quad (1)$$

where A_t is the absorbance at a given time, t, in seconds, A_{inf} is the absorbance at the end of the reaction (t = infinite), A_0 is the absorbance after reductant injection, and k_{obs} is the observed first order rate constant (s^{-1}). All reactions to determine k_{obs} were performed in triplicate. The triplicate values are averaged and divided by the relevant substrate concentration (6 mM) to obtain a second order H-atom transfer rate (k_H, $M^{-1}s^{-1}$) or D-atom transfer rate (k_D), and kinetic isotope effect (KIE) values are calculated from division of k_H by k_D. The KIE value are then treated logarithmically, and plotted against 1/RT, where R is the gas constant and T is the relevant temperature in K. The obtained slope reveals the KIE on activation energies (ΔE_a), and the y-intercept reveals impacts on the Arrhenius prefactor ($\ln(A_H/A_D)$).

General procedure for performing pseudo-first-order reaction kinetics

Pseudo-first-order reaction conditions were used to establish the rate expression for the reaction between the POV, **1-$V_6O_8^{2-}$** or **2-$V_6O_7^{1-}$,** the H-atom transfer reagent, H_2Phen in DMF. Using a UV-vis spectrophotometer with temperature controls set to 298 K, reactions between cluster and excess H_2Phen (10–16.67 eq.) were tracked by monitoring the absorbance at 1025 nm (1000 nm for **2-$V_6O_7^{1-}$**) over the reaction coordinate. Final reductant concentrations were varied from 6–10 mM, with a constant concentration of cluster of 0.6 mM. Samples of cluster stock solutions in DMF were loaded in a long-necked quartz cuvette and sealed with a rubber septum and sealed with electrical tape before removing from the glovebox. In a 1 mL syringe (21G needle), a sample of reductant stock solution was measured prior to removal from the glovebox. After equilibrating to 298 K in the spectrophotometer, data acquisition began, and the reductant solution was forcefully injected to ensure efficient sample mixing, as well as stirring during the reaction. Upon the completion of the reaction, the plot of absorbance over time was fit to the equation 1 by least squares fitting. All reactions to determine k_{obs} were performed in triplicate. The good fit found for reaction curves indicated that the rate expression is first order with respect to reductant concentration (Fig. S5). Plotting k_{obs} as a function of reductant concentration generated a linear plot, meaning that the reaction rate expression is second order overall, such that:

$$\text{rate} = k[\textbf{POV}][H_2\text{Phen}] \quad (2)$$

The slopes of the resultant k_{obs} *vs.* [H_2Phen] plots were normalized for the two possible sites where the H-atom can transfer to in H_2Phen, in order to determine the second order rate constant, k_{PCET} (M^{-1} s^{-1}). With no observed induction period in the pseudo-first order kinetics traces, the *y*-intercept was held at the origin in all cases. The reported errors are the first significant figure of the difference between the determined slope and the confidence interval maximum (95%). To determine the deuterium kinetic isotope effect (KIE), analogous pseudo-first order reactions were performed under identical conditions, using the deuterium-labelled substrate D_2Phen and the relevant POV

(Figures S2, S9 and S11). Uncertainties associated with KIE was determined by accounting for 10% of the average value.

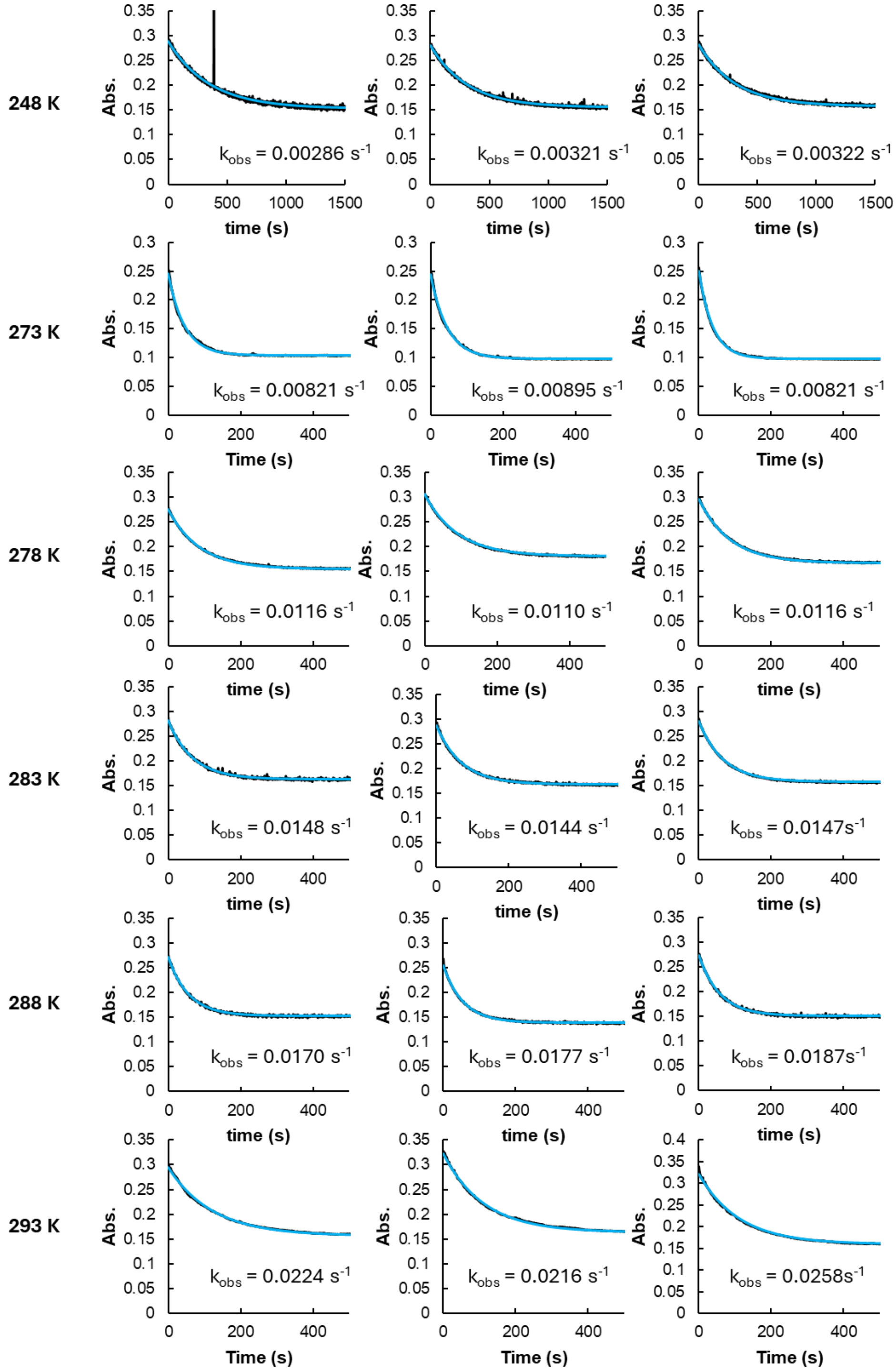

Figure S1. Variable temperature (248-293 K) kinetic traces of **1-$V_6O_8^{2-}$** (0.6 mM) and excess H_2Phen (6.0 mM) in MeCN, monitored at 1025 nm. Temperatures are listed to the left of triplicate trials, and k_{obs} are listed as insets for each trial.

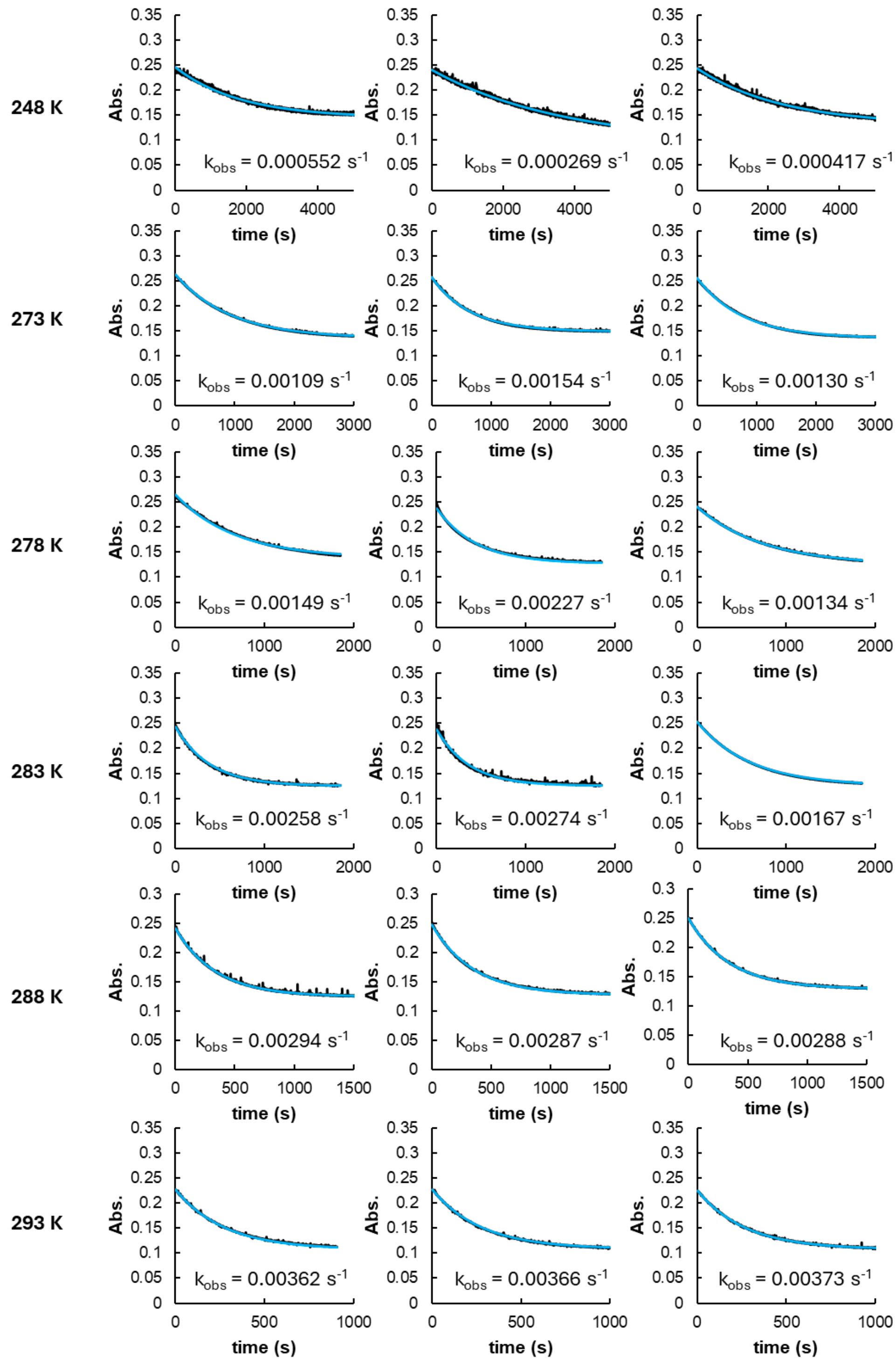

Figure S2. Variable temperature (248-293 K) kinetic traces of **1-$V_6O_8^{2-}$** (0.6 mM) and excess D_2Phen (6.0 mM) in MeCN, monitored at 1025 nm. Temperatures are listed to the left of triplicate trials, and kobs are listed as insets for each trial.

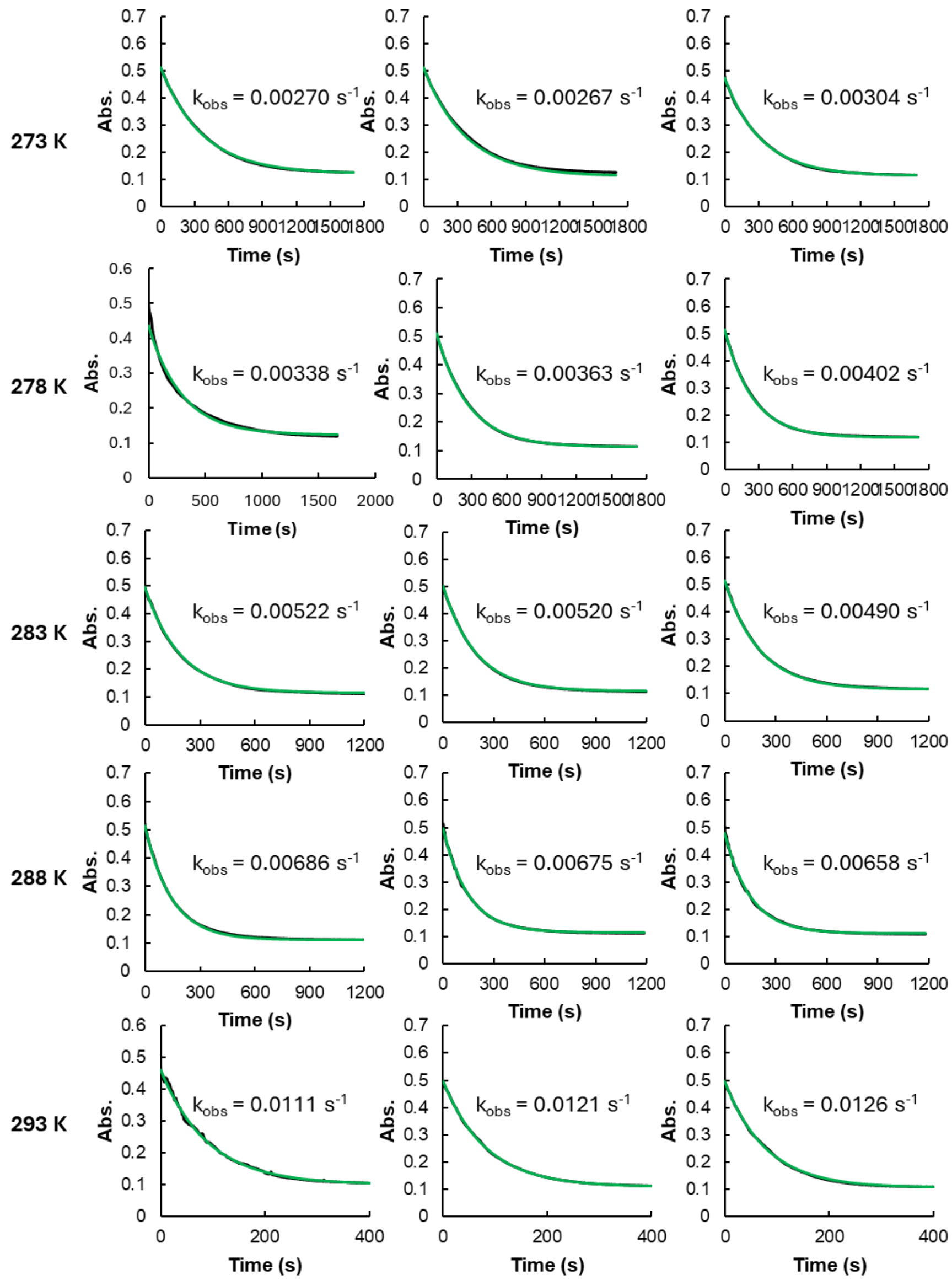

Figure S3. Variable temperature (273-293 K) kinetic traces of **2-$V_6O_7^{1-}$** (0.6 mM) and excess D_2Phen (6.0 mM) in MeCN, monitored at 1025 nm. Temperatures are listed to the left of triplicate trials, and kobs are listed as insets for each trial.

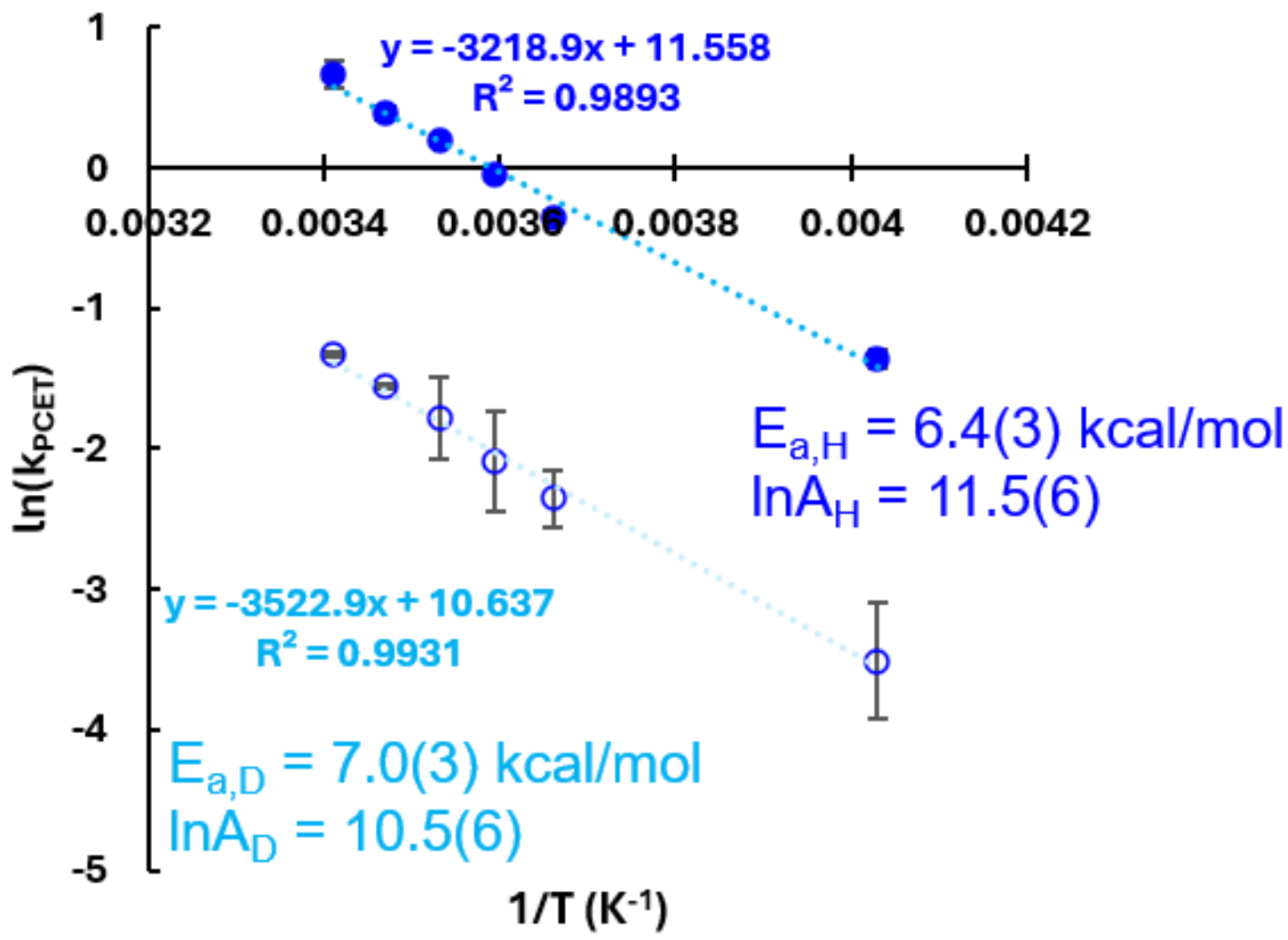

Figure S4. Arrhenius plot of PCET from H_2Phen (solid circles, dark blue) and D_2Phen (hollow circles, light blue) to **1-$V_6O_8^{2-}$** in MeCN from -25 to 25 °C, with activation energies and lnA values included in the insets.

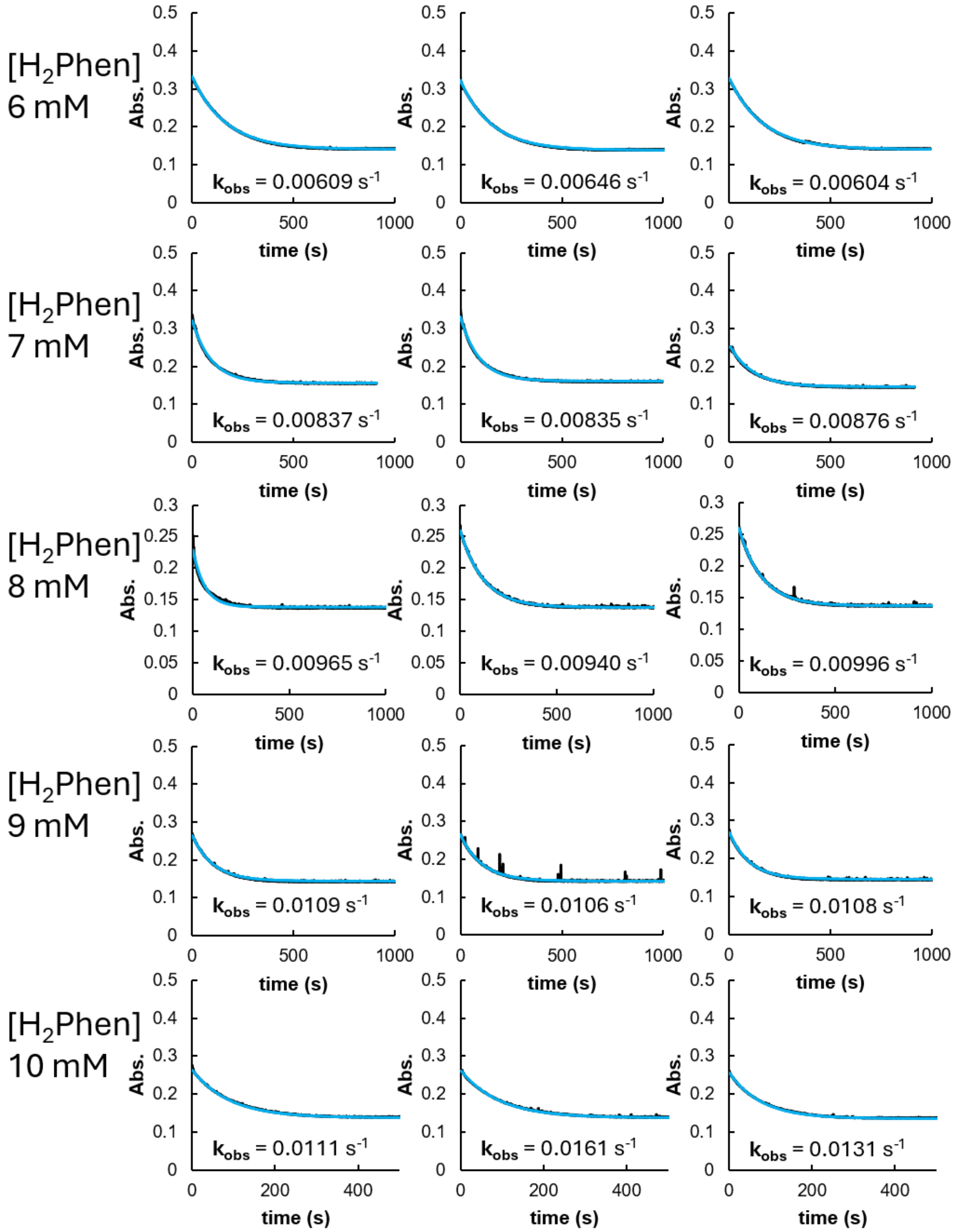

Figure S5. Psuedo-first order kinetic traces of **$1\text{-}V_6O_8^{2-}$** (0.6 mM) and excess H_2Phen (6-10 mM) in DMF at 25 °C, monitored at 1025 nm. Concentrations are listed to the left of triplicate trials and k_{obs} are listed as insets for each trial.

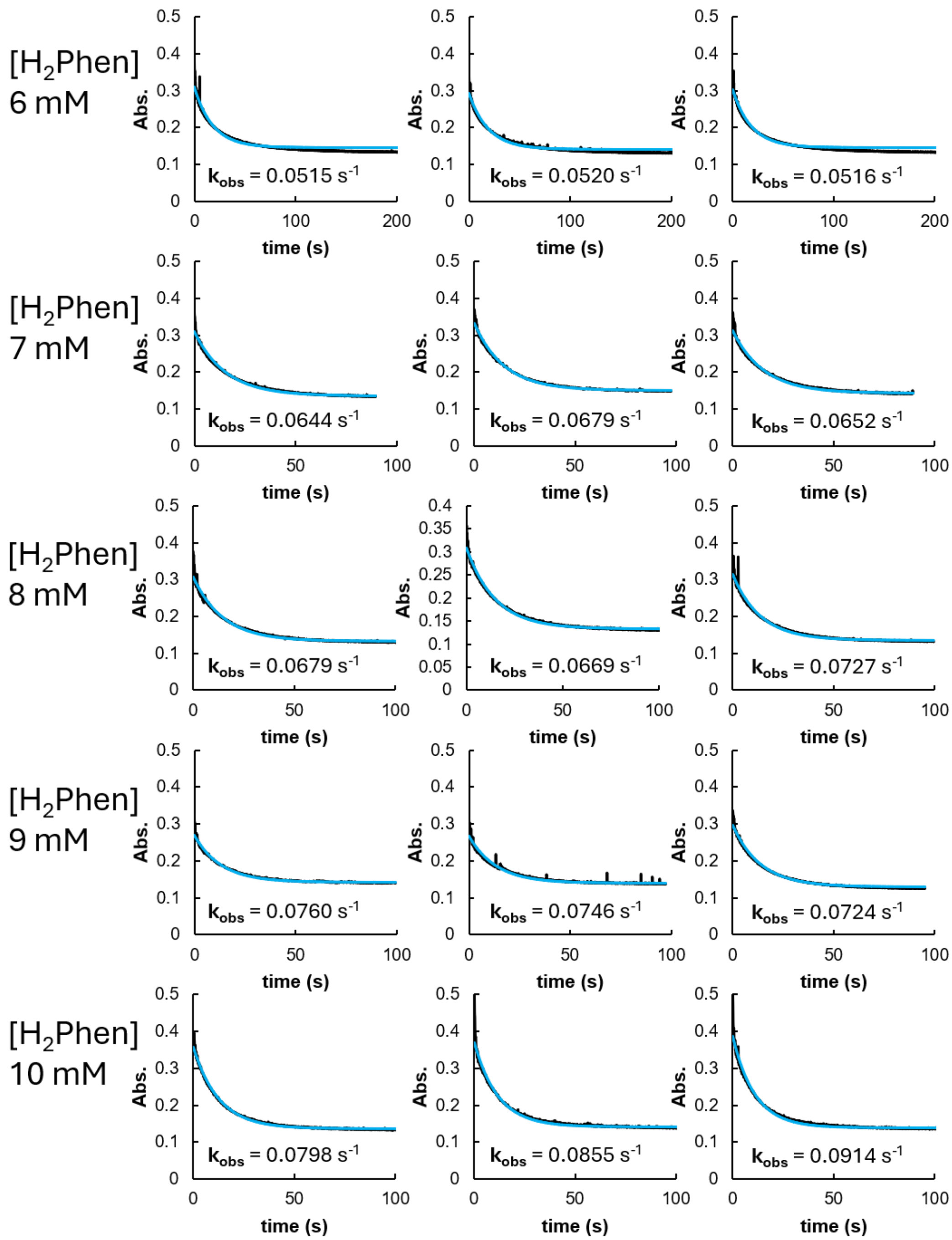

Figure S6. Psuedo-first order kinetic traces of **$2\text{-}V_6O_7^{1-}$** (0.6 mM) and excess H_2Phen (6-10 mM) in DMF at 25 °C, monitored at 1000 nm. Concentrations are listed to the left of triplicate trials and k_{obs} are listed as insets for each trial.

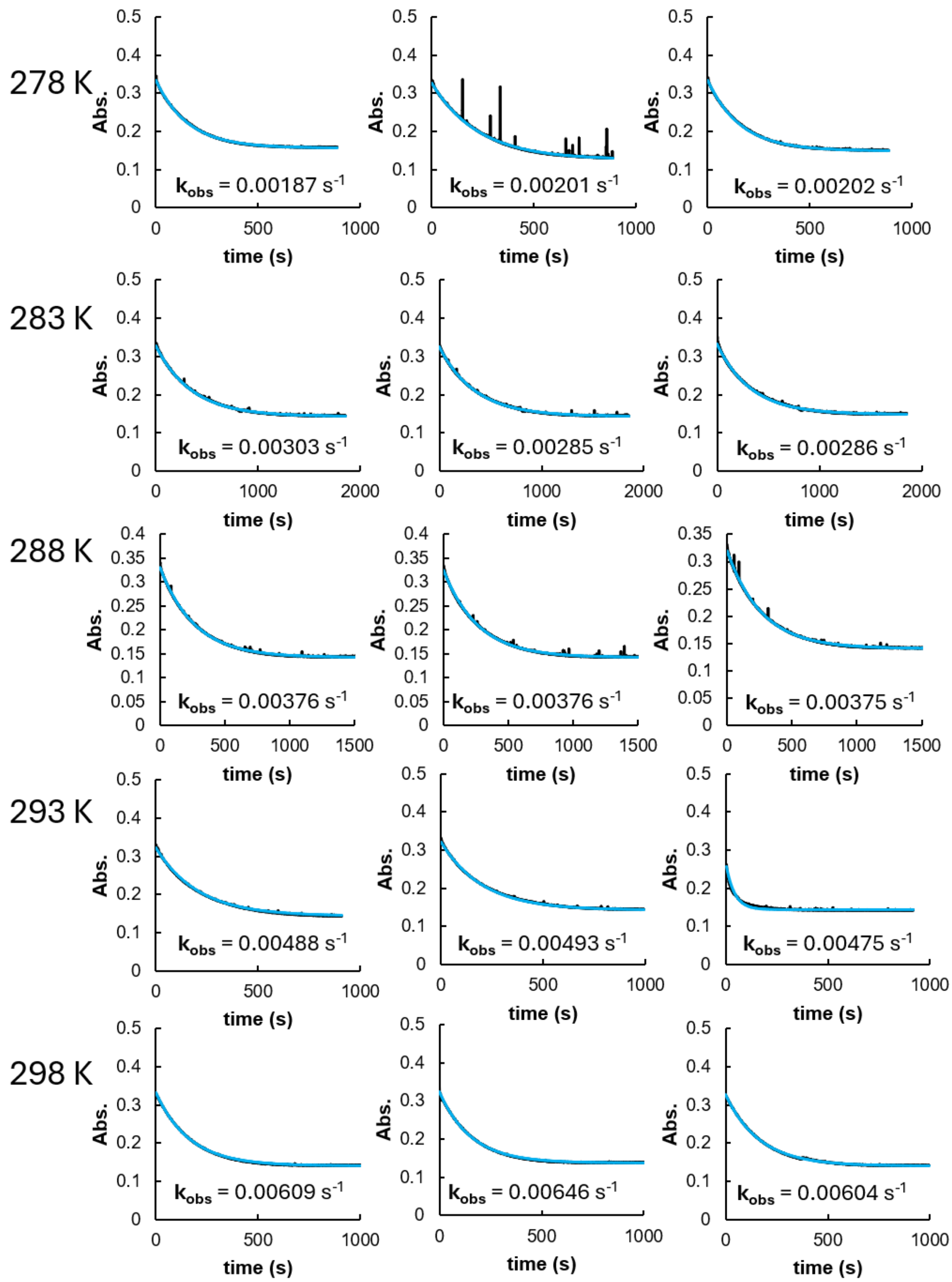

Figure S7. Variable temperature (278-298 K) kinetic traces of **1-$V_6O_8^{2-}$** (0.6 mM) and excess H_2Phen (6 mM) in DMF, monitored at 1025 nm. Temperatures are listed to the left of triplicate trials, and k_{obs} are listed as insets for each trial.

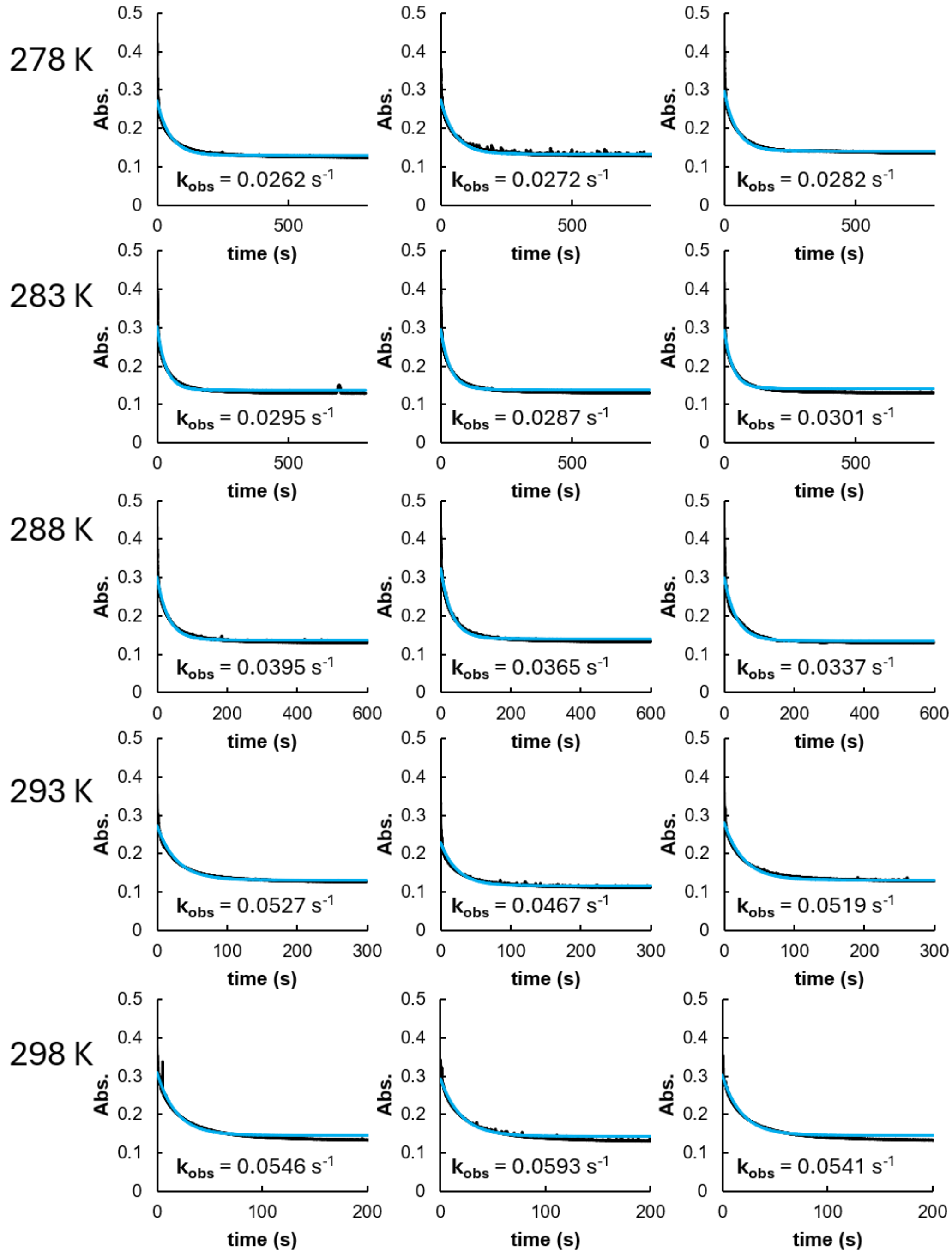

Figure S8. Variable temperature (278-298 K) kinetic traces of **2-$V_6O_7^{1-}$** (0.6 mM) and excess H_2Phen (6 mM) in DMF, monitored at 1000 nm. Temperatures are listed to the left of triplicate trials, and k_{obs} are listed as insets for each trial.

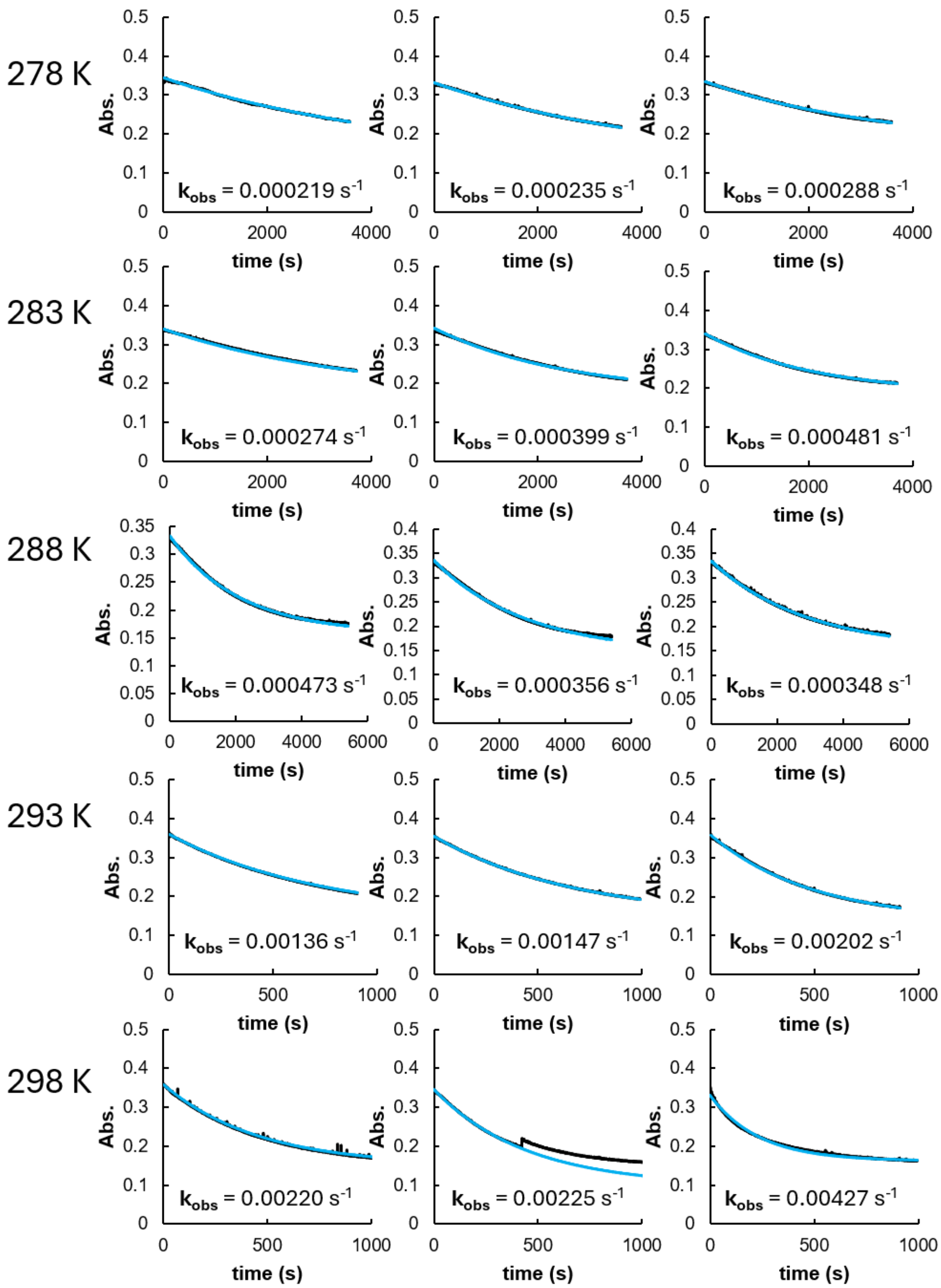

Figure S9. Variable temperature (278-298 K) kinetic traces of **1-$V_6O_8^{2-}$** (0.6 mM) and excess D_2Phen (6 mM) in DMF, monitored at 1025 nm. Temperatures are listed to the left of triplicate trials, and *k_{obs}* are listed as insets for each trial.

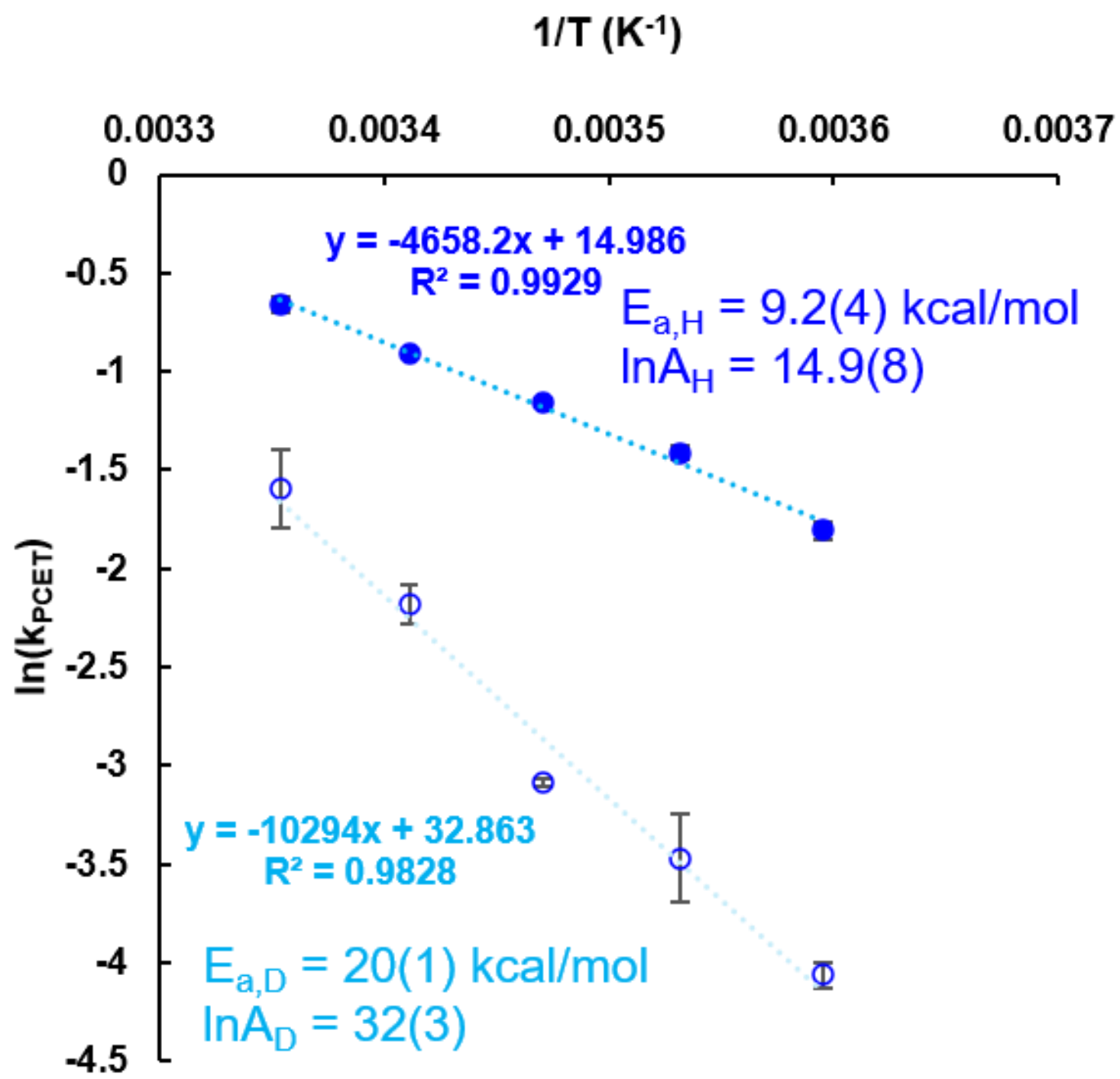

Figure S10. Arrhenius plot of PCET from H_2Phen (solid circles, dark blue) and D_2Phen (hollow circles, light blue) to **1-$V_6O_8^{2-}$** in DMF from 5 to 25 °C, with activation energies and lnA values included in the insets.

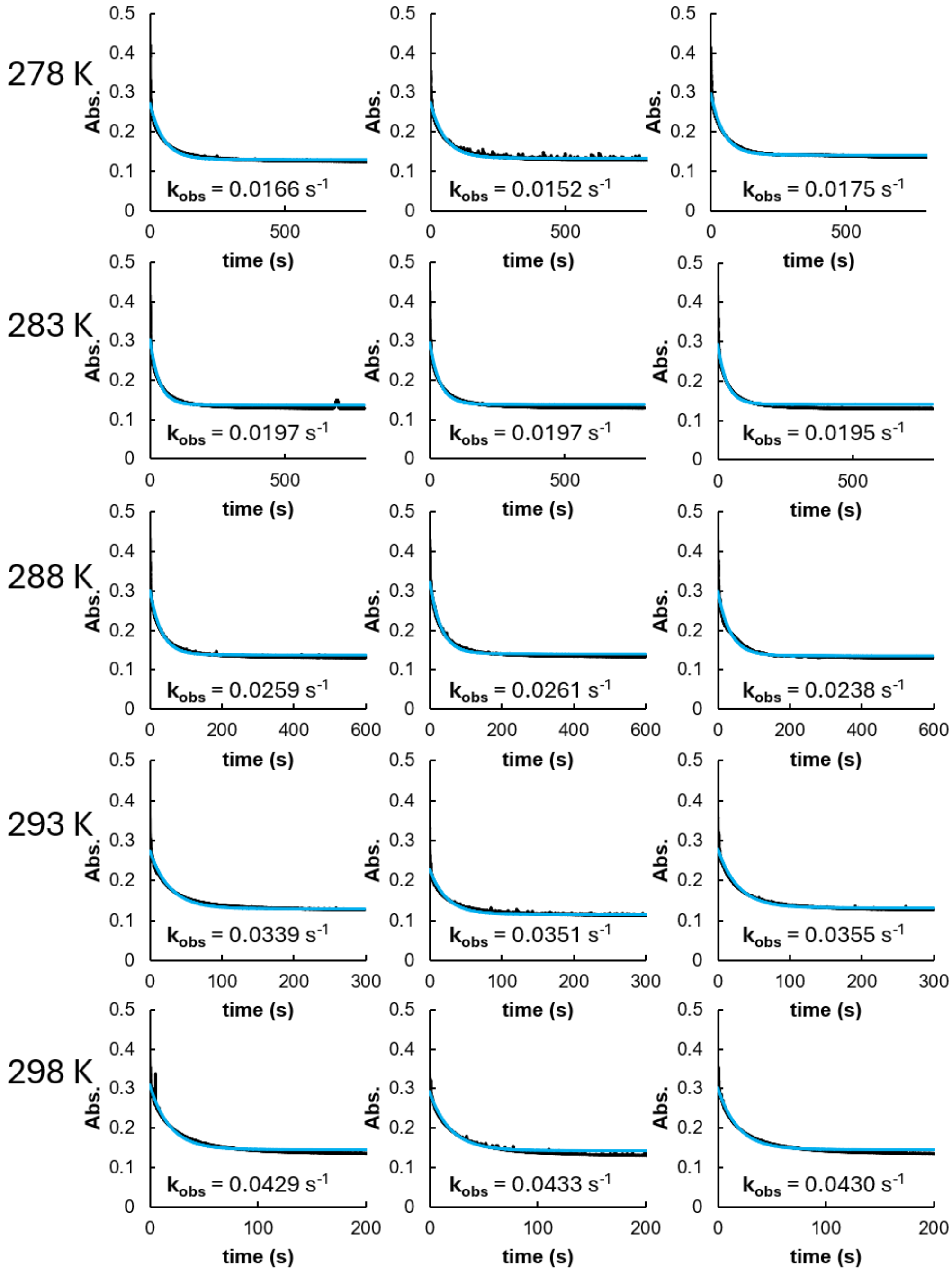

Figure S11. Variable temperature (278-298 K) kinetic traces of **2-$V_6O_7^{1-}$** (0.6 mM) and excess D_2Phen (6 mM) in DMF, monitored at 1000 nm. Temperatures are listed to the left of triplicate trials, and k_{obs} are listed as insets for each trial.

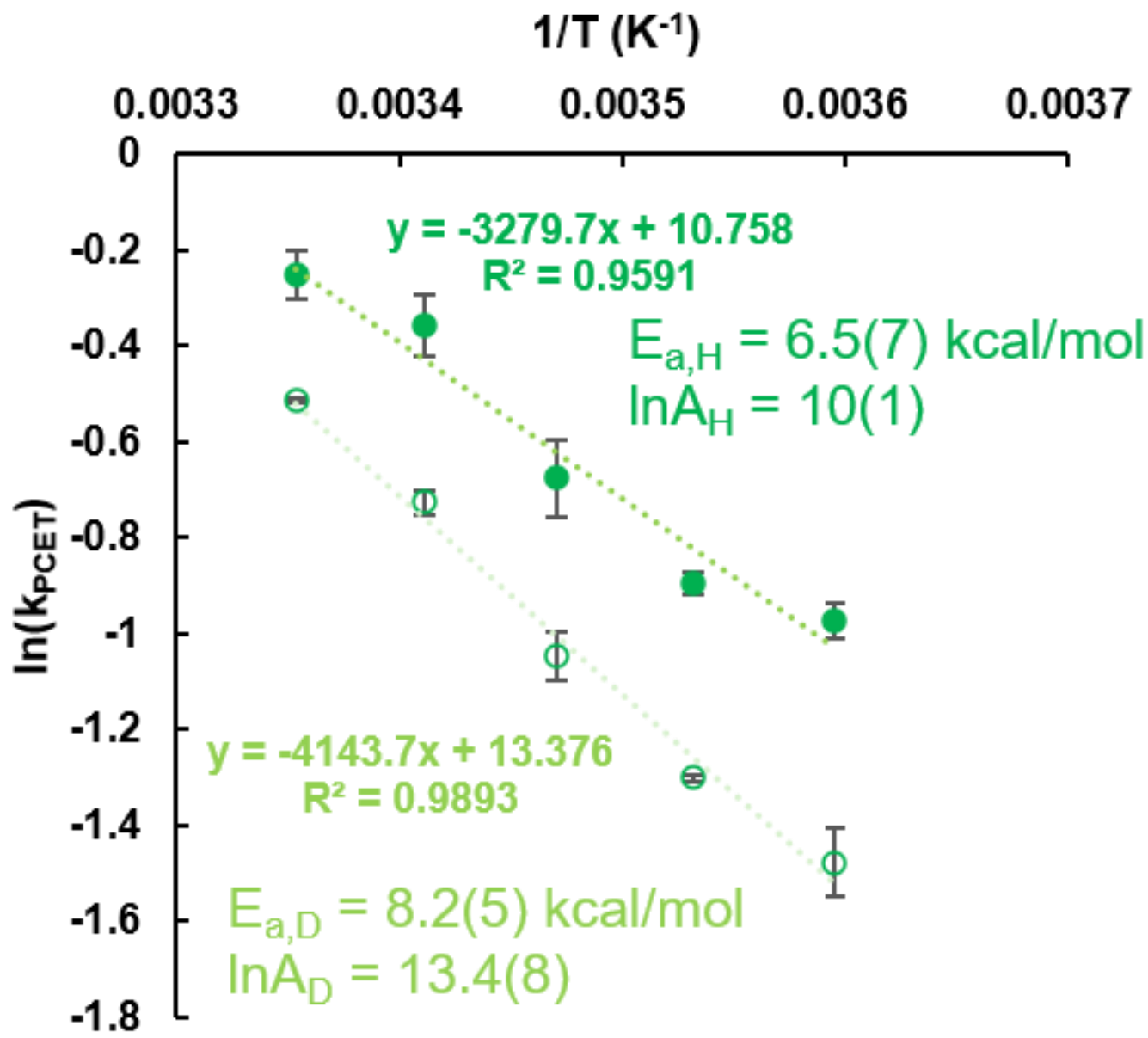

Figure S12. Arrhenius plot of PCET from H_2Phen (solid circles, green) and D_2Phen (hollow circles, light green) to **2-$V_6O_7$$^{1-}$** in DMF from 5 to 25 °C, with activation energies and lnA values included in the insets.

References

(1) Marinho, T. V.; Schreiber, E.; Garwick, R. E.; Brennessel, W. W.; Matson, E. M. Manipulating Ligand Density at the Surface of Polyoxovanadate-Alkoxide Clusters. *Inorg. Chem.* **2023**, *62* (38), 15616-15626. DOI: 10.1021/acs.inorgchem.3c02314.
(2) Daniel, C.; Hartl, H. Neutral and Cationic VIV/VV Mixed-Valence Alkoxo-polyoxovanadium Clusters [V6O7(OR)12]n+ (R = −CH3, −C2H5): Structural, Cyclovoltammetric and IR-Spectroscopic Investigations on Mixed Valency in a Hexanuclear Core. *Journal of the American Chemical Society* **2005**, *127* (40), 13978-13987. DOI: 10.1021/ja052902b.
(3) Lee, J.; Shizu, K.; Tanaka, H.; Nakanotani, H.; Yasuda, T.; Kaji, H.; Adachi, C. Controlled emission colors and singlet–triplet energy gaps of dihydrophenazine-based thermally activated delayed fluorescence emitters. *Journal of Materials Chemistry C* **2015**, *3* (10), 2175-2181, 10.1039/C4TC02530J. DOI: 10.1039/C4TC02530J.
(4) Schreiber, E.; Fertig, A. A.; Brennessel, W. W.; Matson, E. M. Oxygen-Atom Defect Formation in Polyoxovanadate Clusters via Proton-Coupled Electron Transfer. *Journal of the American Chemical Society* **2022**, *144* (11), 5029-5041. DOI: 10.1021/jacs.1c13432.
(5) Fulmer, G. R.; Miller, A. J. M.; Sherden, N. H.; Gottlieb, H. E.; Nudelman, A.; Stoltz, B. M.; Bercaw, J. E.; Goldberg, K. I. NMR Chemical Shifts of Trace Impurities: Common Laboratory Solvents, Organics, and Gases in Deuterated Solvents Relevant to the Organometallic Chemist. *Organometallics* **2010**, *29* (9), 2176-2179. DOI: 10.1021/om100106e.
(6) Peter, C. Y. M.; Schreiber, E.; Proe, K. R.; Matson, E. M. Surface ligand length influences kinetics of H-atom uptake in polyoxovanadate-alkoxide clusters. *Dalton Transactions* **2023**, *52* (43), 15775-15785, 10.1039/D3DT02074F. DOI: 10.1039/D3DT02074F.
